# Deactivation of 6-Aminocoumarin Intramolecular Charge Transfer Excited State through Hydrogen Bonding

**DOI:** 10.3390/ijms150916628

**Published:** 2014-09-19

**Authors:** Ewa Krystkowiak, Krzysztof Dobek, Andrzej Maciejewski

**Affiliations:** 1 Faculty of Chemistry, Adam Mickiewicz University in Poznań, Umultowska 89b, Poznań 61-614, Poland; E-Mail: iwonam@amu.edu.pl; 2 Faculty of Physics, Adam Mickiewicz University in Poznań, Umultowska 85, Poznań 61-614, Poland; E-Mail: dobas@amu.edu.pl; 3 Center for Ultrafast Spectroscopy, Adam Mickiewicz University in Poznań, Umultowska 85, Poznań 61-614, Poland

**Keywords:** 6-aminocoumarin, solvatochromism, hydrogen bonding, donor-acceptor system, electronic excited state, energy gap law

## Abstract

This paper presents results of the spectral (absorption and emission) and photophysical study of 6-aminocoumarin (6AC) in various aprotic hydrogen-bond forming solvents. It was established that solvent polarity as well as hydrogen-bonding ability influence solute properties. The hydrogen-bonding interactions between S_1_-electronic excited solute and solvent molecules were found to facilitate the nonradiative deactivation processes. The energy-gap dependence on radiationless deactivation in aprotic solvents was found to be similar to that in protic solvents.

## 1. Introduction

The majority of dyes have, besides aromatic moieties, numerous heteroatoms (e.g., N, O, S) and chemical bonds including protic hydrogen atoms (–OH, –NH_2_, –NHR) in their molecular structure. Therefore, these molecules can interact with solvent molecules not only non-specifically but they also form hydrogen bonds of donor as well as acceptor character. Because the electronic excitation of the dye molecule leads to significant changes in electronic density, the energy of hydrogen bonds also changes as a result of this process. In the dye molecule, which is usually complex, a great number of hydrogen bonds of donor and acceptor character can be formed. Thus a description of the solvent influence on the absorption and emission spectra as well as on the photophysical and photochemical properties of a dye is very complicated. Therefore the results of similar studies for less complex probe molecules having in their structure the same chromophores as those present in the studied dye molecules can be very helpful for a better understanding of the processes involved.

As we have shown [1,2,3,4,5] for hydrogen-bond forming probes, correct and careful choice of solvents plays a very important role in the spectral (particularly solvatochromic), as well as in photophysical and photochemical studies. The solvent effect can be split into two different types of contributions, namely specific interactions and non-specific interactions. The specific ones are described as localized donor-acceptor interactions involving specific orbitals, acid-base interactions involving hydrogen bonding, charge-transfer interactions, and π–π electron interactions. The non-specific interactions arise from the solvent acting as a dielectric medium [6]. Solvents can be classified in many ways according to their polarity, acidity, electron pair or proton-donating properties, *etc.* [7]. The classification of solvents according to their polarity as well as hydrogen-bonding ability is most often performed on the basis of the criteria proposed by Kamlet and Taft [8,9,10] and Catalan *et al.* [6,11,12], both based on spectroscopic measurements. The solvents capable of forming hydrogen bonds have been classified as hydrogen-bond acceptors and hydrogen-bond donors. The hydrogen-bond acceptor property of a solvent depends on its ability to accept a hydrogen atom from a solute to form a hydrogen bond (β in Kamlet-Taft’s and SB in Catalan scale), and the hydrogen-bond donor property depends on its ability to donate a hydrogen atom to form a hydrogen bond with a solute (α in Kamlet-Taft’s and SA in Catalan scale). The α scale was selected to extend from 0.0 for non-hydrogen-bond donor solvents to ~1.96 for hexafluoroisopropanol and the β scale from 0.0 for cyclohexane to ~1.0 for hexamethylphosphoric acid triamide [13]. The protic solvents most often used in solvatochromic studies (alcohols, diols, water and acids) have properties of both hydrogen-bond donors and acceptors. In solvatochromic experiments the shifts in absorption and emission maxima depend not only on the changes in the energy of non-specific interactions but also on those in the energy of hydrogen bonds made between the probe and the solvent molecules [1,14]. Therefore of importance in these studies is to use the solvents having only hydrogen-bond donor or only hydrogen-bond acceptor properties and also the solvents interacting only non-specifically with the solute. In reference [1] we proposed the procedure allowing the determination of the changes in hydrogen bond energy as a result of electronic transition on the basis of experimental absorption and emission solvatochromic studies.

**Figure 1:**
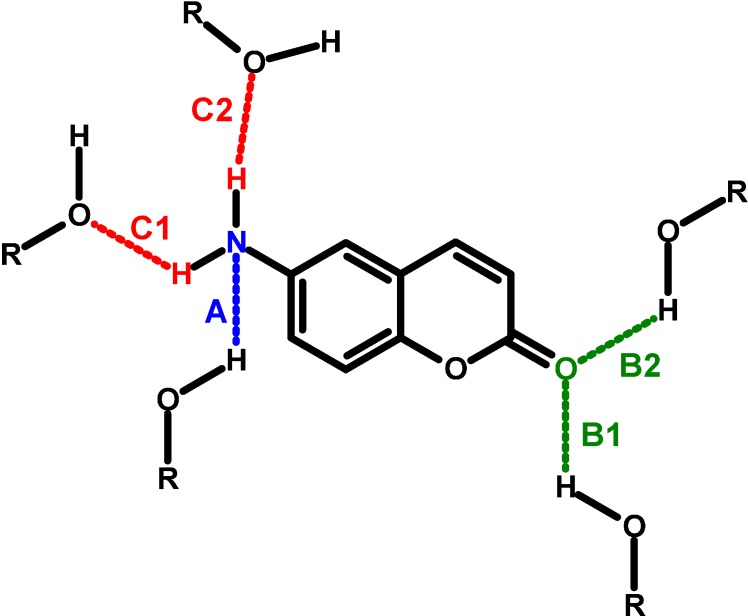
Illustration of different types of intermolecular hydrogen bonds between 6-aminocoumarin (6AC) and solvent molecules.

As a probe in our study we have chosen a coumarin dye 6-aminocoumarin, 6AC, which belongs to the donor-acceptor compounds as it contains a donor amine group and an acceptor carbonyl group. Both these chromophores are often present in dye molecules. In the 6AC molecule, an amino group is substituted at position 6 of the 1,2-benzopyrone moiety. Therefore, in contrast to 7-aminocoumarins (often used as fluorescence probes [15,16,17,18,19,20,21,22], chemosensors [23,24,25] in biological and biomedical sciences [26,27,28,29] and in dye lasers [30,31,32,33]), the donor and acceptor groups are arranged along the same molecular axis and no other substituent groups (e.g., –CH_3_, –CF_3_) are present in the molecule. In general, aromatic carbonyl compounds with amino group, such as aminocoumarins, can form intermolecular hydrogen bonds with solvents at different sites both in the ground and excited states [3,4,5,34,35,36,37,38]. In 6AC there are at least three sites of hydrogen bond formation with a solvent molecule as shown in Figure 1: the hydrogen bond between the lone pair of electrons from the nitrogen atom of the amino group and the hydrogen atom from the solvent molecule (A type hydrogen bond), the carbonyl oxygen atom and the hydrogen atom from the solvent molecule (B type hydrogen bonds), and the hydrogen atoms from the amino group and the electronegative heteroatom from the solvent molecule (C type hydrogen bonds). Recently we have investigated the spectral and photophysical properties of 6AC in exclusively non-specifically interacting solvents of different polarities [4] and in protic solvents [5]. To complete spectral and photophysical characterisation of 6AC, a similar study is necessary in the solvents which form only the hydrogen bonds of acceptor character with the 6AC molecule (Kamlet-Taft’s solvatochromic parameters β > 0 and α = 0).

The main aim of our study was to determine the effect of intermolecular hydrogen bonds of C type (Figure 1) on the spectral and photophysical properties of 6AC in the ground (S_0_) and the first excited singlet states (S_1_). We have used in our study the range of solvents having only hydrogen-bond acceptor properties. This means that 6AC and the solvent molecules can form complexes via C type hydrogen bond only. Simple and sufficiently rigid structure of the probe molecule should ensure the lack of significant changes in geometry after electronic excitation process. This fact will significantly facilitate the determination of 6AC molecule properties after excitation. We have used in our study a few solvents, whose hydrogen-bond formation ability changes in a wide range (0.25 ≤ β ≤ 1.0, where β Kamlet–Taft’s solvatochromic parameter) but they are characterised by similar energy of non-specific solute-solvent interaction (similar value of solvent polarity function, *f*(ε, *n*^2^); defined in Table 1 footnote). It should permit determination of the influence of hydrogen bond energy on the formation of the stable solute-solvent complexes in the ground and excited electronic states. Additionally, we have studied 6AC in the solvents of hydrogen-bond acceptor properties differing in polarity.

**Table 1:** Absorption and emission spectral properties of 6AC (1 × 10^−4^ mol dm^−3^) in aprotic hydrogen-bond forming solvents and in 1-chloro-*n*-propane (non-specifically interacting solvent).

Solvent	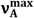 (cm^−1^)	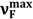 (cm^−1^)	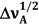 (cm^−1^)	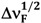 (cm^−1^)	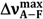 (cm^−1^)	ε( 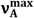 ) (mol^−1^·dm^3^·cm^−1^)	*f*(ε, *n*^2^)	ε *^a^*	*n* *^a^*	α *^a^*	β *^a^*
1-Chloro-*n*-hexadecane *^b^*	27,250	21,030	4780	4350	6220	−	0.110	3.70	1.450	0.00	0.00
1-Chloro-*n*-decane *^b^*	27,250	20,750	4640	4370	6500	2860	0.145	4.58	1.438	0.00	0.00
1-Chloro-*n*-octane	27,250	20,650	4600	4350	6600	3240	0.160	5.05	1.430	0.00	0.00
1-Chloro-*n*-hexane	27,250 *^c^*	20,490	4650	4320	6760	3270	0.184	6.10	1.419	0.00	0.00
1-Chloro-*n*-butane	27,250 *^c^*	20,400	4650	4300	6850	3350	0.209	7.39	1.400	0.00	0.00
1-Chloro-*n*-propane *^b^*	27,250	20,300	4600	4400	6950	3450	0.226	8.59	1.386	0.00	0.00
Acrylonitrile	26,950	18,470	4660	4270	8480	2850	0.287	33.00	1.388	0.00	0.25
Propionitrile *^b^*	26,880	18,810	4750	4340	8070	2750	0.292	28.26	1.363	0.00	0.37
DBE	26,950	20,070	4620	4330	6880	2820	0.096	3.08	1.397	0.00	0.46
THF	26,670	19,430	4840	4480	7240	2930	0.210	7.58	1.405	0.00	0.55
DMF	26,100	17,850	4810	4220	8250	2800	0.275	36.71	1.428	0.00	0.69
DMSO	25,910 *^c^*	17,500	4800	4180	8410	2860	0.264	46.45	1.477	0.00	0.76
HMPA	25,250	17,550	5200	4170	7700	2340	0.261	29.30	1.457	0.00	1.00

ε (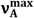), molar extinction coefficient; *n*, refraction coefficient; ε, dielectric constant; α, Kamlet-Taft’s solvatochromic parameter related to hydrogen-bond donating ability; β, Kamlet-Taft’s solvatochromic parameter related to hydrogen-bond accepting ability; *f*(ε, *n*^2^) = (ε − 1)/(2ε + 1) – (*n*^2^ − 1)/(2*n*^2^ + 1); *^a^* From reference [13]; *^b^* From reference [4]. A typing error in 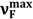 for 6AC in 1-chloro-*n*-propane in references [4,5] was noticed; *^c^* From reference [3]; DBE, di-*n*-butyl ether; HMPA, hexamethylphosphoramide; THF, tetrahydrofuran; DMSO, dimethyl sulphoxide; and DMF, *N*,*N*-dimethylformamide.

The results of our present study, together with those obtained for 6AC recently [4,5], can be used in the discussion of the properties of many dyes in solvents of various polarities and hydrogen-bond formation abilities and also various complex systems of great practical importance, e.g., micellar systems, ionic liquids, and cyclodextrins.

## 2. Results and Discussion

### 2.1. Spectral Properties of 6AC in Aprotic Hydrogen-Bond Forming Solvents

**Figure 2:**
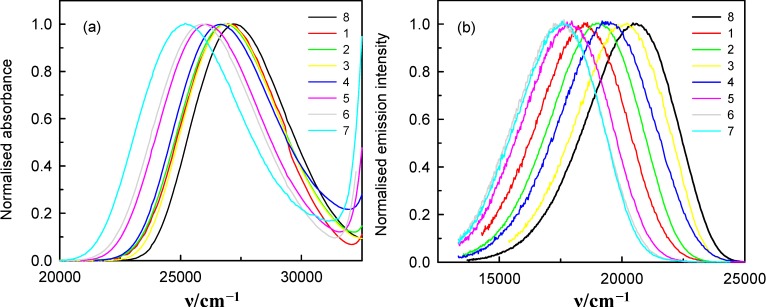
Normalised absorption (**a**) and fluorescence (**b**) spectra of 6AC in (1) acrylonitrile; (2) *^b^* propionitrile; (3) di-*n*-butyl ether (DBE); (4) tetrahydrofuran (THF); (5) *N*, *N*-dimethylformamide (DMF); (6) *^a^* dimethyl sulphoxide (DMSO); (7) hexamethylphosphoramide (HMPA); and (8) *^a,b^* 1-chloro-*n*-propane. *^a^* From reference [3]. *^b^* From reference [4].

The absorption and steady-state emission spectra measurements were performed for 6AC in various aprotic hydrogen-bond forming solvents. They are presented in Figure 2, together with those in 1-chloro-*n*-propane (the solvent, which interacts only non-specifically with 6AC [3,4]).

**Figure 3:**
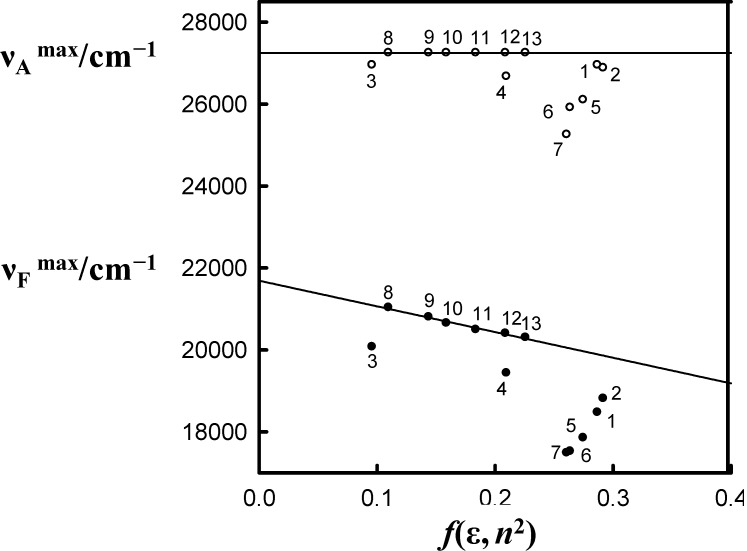
The solvatochromic plot of 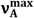 (circles) and 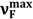 (filled circles) as a function of *f*(ε, *n*^2^) (see Table 1 for definition) for 6AC in (1) acrylonitrile; (2) *^b^* propionitrile; (3) DBE; (4) THF; (5) DMF; (6) *^a^* DMSO; (7) HMPA; (8) *^a,b^* 1-chloro-*n*-hexadecane; (9) *^a,b^* 1-chloro-*n*-decane; (10) *^a,b^* 1-chloro-*n*-octane; (11) *^a,b^* 1-chloro-*n*-hexane; (12) *^a,b^* 1-chloro-*n*-butane; and (13) *^a,b^* 1-chloro-*n*-propane. *^a^* From reference [3], *^b^* From reference [4].

As can be seen in Figure 2a and Table 1, the long-wavelength absorption band maxima, 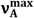, in the spectra of 6AC in all solvents, which can form hydrogen bonds of acceptor character only, shift towards longer wavelength with respect to that in the spectrum of 6AC in 1-chloro-*n*-propane. The shapes of the long-wavelength band (Figure 2a) do not differ significantly in the spectra taken for all solvents used, but FWHM (full width at half maximum) in the absorption spectra, 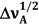, increases slightly, while the batochromic shift increases significantly with increasing β Kamlet-Taft’s solvent parameter (Table 1). Similarly to 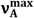 all 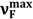 in the fluorescence spectra of 6AC in aprotic hydrogen-bond forming solvents (see Figure 2b and Table 1) are shifted towards longer wavelength with respect to that in the spectrum of 6AC in 1-chloro-*n*-propane. The shape of the fluorescence band (Figure 2b) and the FWHM value of the fluorescence spectra of 6AC, 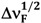, are practically the same in all solvents used, similarly as that of the absorption spectra, but 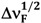 is lower than that for 6AC in 1-chloro-*n*-propane. No influence of the excitation wavelength, λ_exc_, on the position and shape of the fluorescence spectrum was noted, similarly as reported earlier in references [4,5,39]. Very large Stokes shifts, in comparison with those of 7-aminocoumarins (e.g., [40,41]), have been observed between the absorption and fluorescence maxima (as reported earlier in reference [39]). This indicates that the changes in energy of solute–solvent specific (hydrogen bond formation) and non-specific interactions have a great influence on the static spectroscopic properties of 6AC. Table 1 lists the absorption and fluorescence maxima (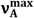 and 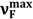, respectively), FWHM values of the absorption and fluorescence (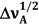 and 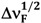, respectively) and the Stokes shifts, 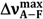 = 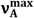 − 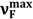, of 6AC in different solvents. It also gives some of solvents properties and the solvent polarity function, *f*(ε, *n*^2^), values. The dependence of solvatochromic plots of the 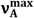 and 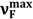 on *f*(ε, *n*^2^) of 6AC in aprotic hydrogen-bond forming and in non-specifically interacting solvents are given in Figure 3.

As shown in Figure 3 the 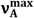 and 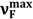 , values for 6AC in aprotic hydrogen-bond forming solvents deviate from the linear correlations observed in 1-chloro-*n*-alkanes. This is a result of C type hydrogen bond formation between the solute and solvent molecules, which are stronger in the excited S_1_ state than in the ground S_0_ state [3]. Results of the theoretical study performed by Yang *et al.* [42] show that upon the S_0_→S_1_ excitation of the 6AC molecule, the electron density from the amino group in the ground S_0_ state disappears completely, and the electron density is localized over the entire molecule except the amino group. Therefore, the energies of hydrogen bonds formed by the solvent molecules with two hydrogen atoms of the amino group of 6AC should be significantly increased after excitation to the S_1_ state. Similarly to the 6AC molecule, the electron density redistribution from the amino group to the carbonyl group upon the S_0_→S_1_ excitation was found for the C120 molecule [36,43,44,45] and other coumarin molecules [46,47,48,49]. Moreover, on the basis of absorption and emission solvatochromic data obtained in this study (see Figure 3), as well as the results presented in references [4,5], it can be assumed that for 6AC and its complexes with solvent molecules, electron density on the nitrogen atom from the amino group decreases and that on the oxygen atom from carbonyl group increases in the emitting S_1_ state compared with those in the S_1_ state directly after excitation.

**Figure 4:**
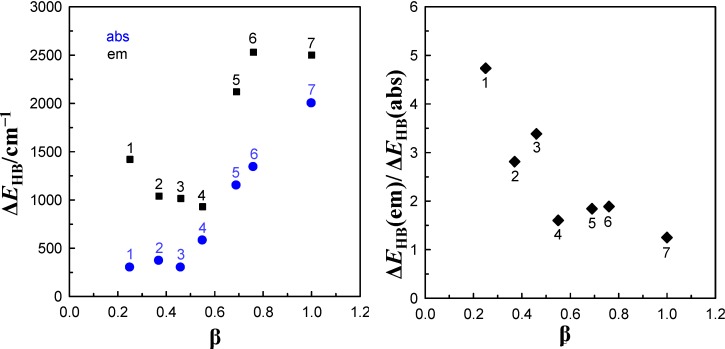
Correlation between the hydrogen bond energy changes, ∆*E*_HB_, as a result of absorption or emission process and the solvent Kamlet-Taft’s hydrogen-bonding accepting parameter, β, for 6AC-(solvent)*_n_*, *n* = 1, 2, complexes: (1) acrylonitrile, (2) propionitrile, (3) DBE, (4) THF, (5) DMF, (6) DMSO, (7) HMPA.

According to the procedure proposed by us in references [1,3,5], the experimental solvatochromic study permits determination of hydrogen bond energy changes, ∆*E*_HB_, as a result of transition between two different electronic states. This procedure is based on an analysis of the solvatochromic dependencies of 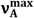 or 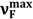 on the polarity function *f*(ε, *n*^2^) of the solvents used. The first step of the procedure required determination of the contribution coming from the nonspecific interactions only using the experimentally observed solvent spectral shifts in the absorption or emission spectra of the solute studied in the several 1-chloro-*n*-alkanes (interacting only non-specifically with solute). The evidence are the straight lines (see Figure 3) obtained as plots of the relation between 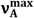 or 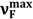 on the *f*(ε, *n*^2^). Therefore it can be assumed that these lines describe the effect of non-specific solute–solvent interactions on the 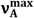 and 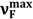 values not only in 1-chloro-*n*-alkanes but also in solvents that make hydrogen bonds with the solute, as long as these solvents satisfy the other assumptions of the Onsager reaction field model of interactions with the solute molecule. The distance between the straight line and the point corresponding to experimental 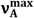 or 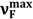 value in a given solvent is a measure of total ∆*E*_HB_ of the hydrogen bonds formed between solute and solvent molecules. The values of ∆*E*_HB_ experimentally obtained from spectral absorption and emission solvatochromic study (corresponding to S_0_→S_1_ and S_1_→S_0_ transitions, respectively) for 6AC-(solvent)*_n_*, *n* = 1, 2, complexes are collected in Table 2. The correlation between ∆*E*_HB_ values and the Kamlet-Taft’s solvatochromic β solvent parameter are presented in Figure 4. As follows from Table 2 and Figure 4 for all 6AC-(solvent)*_n_*, *n* = 1, 2, complexes, the ∆*E*_HB_ values obtained for S_1_→S_0_ emission process are higher than the corresponding ones for S_0_→S_1_ absorption process. The ∆*E*_HB_(em)/∆*E*_HB_(abs) ratio decreases almost linearly with increasing β Kamlet-Taft solvent parameter. The value of ∆*E*_HB_ due to S_0_→S_1_ excitation process determined for 6AC-(DMSO)*_n_* complex is a bit lower than that estimated on the basis of the theoretical study for 6AC by Yang *et al.* [42]. The ∆*E*_HB_ values obtained for 6AC-(DMSO)*_n_* and 6AC-(DMF)*_n_* complexes are also a bit lower than those calculated for C120-(DMSO)_2_ and C120-(DMF)_2_ complexes, respectively [36,45]. Unfortunately, there are no theoretical calculation data concerning S_1_→S_0_ emission process for intermolecular complexes of 6AC or other similar in the structure aminocoumarin derivatives with solvent.

**Table 2:** Hydrogen bond energy changes for 6AC-(solvent)*_n_*, *n* = 1, 2, complexes as a result of S_0_→S_1_ excitation and S_1_→S_0_ emission process determined on the basis of solvatochromic study. All values in cm^−1^.

Solvent	∆*E*_HB_	
	S_0_→S_1_	S_1_→S_0_
Acrylonitrile	300	1420
Propionitrile	370	1040
DBE	300	1015
THF	580	930
DMF	1150	2120
DMSO	1340 *^a^*	2530
HMPA	2000	2500

*^a^* From reference [3].

### 2.2. Hydrogen-Bonded Complexes in Ground S_0_ and Excited S_1_ States

The values of hydrogen bond energy in individual electronic state can be determined only theoretically. Therefore the hydrogen bond energies, *E*_HB_, of C type hydrogen bonds in the S_0_ state formed by the 6AC molecule with one or two HMPA molecules were determined using *ab initio* methods. This solvent was chosen because of the highest β Kamlet-Taft’s parameter value. Estimated *E*_HB_ values in the S_0_ state for the 6AC-(HMPA) complex are 5670 cm^−1^ for C1 bond and 6080 cm^−1^ for C2 bond, while for the 6AC-(HMPA)_2_ complex it is 13,050 cm^−1^. The *E*_HB_ values in the S_0_ state determined by Yang *et al.* [42] for the 6AC-(DMSO)_2_ complex were 2852 cm^−1^ for the C1 bond and 2362 cm^−1^ for the C2 bond. According to the results presented by Zhou *et al.* [36] for C120-(solvent)_2_ complexes, the hydrogen bond energy in the S_0_ state equals 3998 cm^−1^ for the C1 bond and 3217 cm^−1^ for the C2 bond for C120-(DMSO)_2_ and 2394 cm^−1^ for the C1 bond and 2058 cm^−1^ for the C2 bond for C120-(DMF)_2_ complexes, respectively. Assuming a similar relation in C1 and C2 bond energies for 6AC in DMSO and in DMF, one can estimate the energy of ~1700 cm^−1^ for the 6AC-(DMF)_2_ C1 bond and ~1500 cm^−1^ for the C2 bond.

Taking into account these hydrogen bond energy values estimated theoretically for some complexes in the ground S_0_ state and the Kamlet-Taft’s β parameter values for all aprotic hydrogen-bond forming solvents used in this study, it is possible to propose the dominant species, which most probably exists in this state in the solution of 6AC in each solvent. High values of *E*_HB_ clearly show that in the case of DMSO and HMPA, the significant majority of 6AC molecules must form in the ground S_0_ state 6AC-(solvent)_2_ complexes. In the case of DMF, the 6AC molecules can form 6AC-(solvent)_2_ complexes as well as 6AC-(solvent)_1_ complexes. Following β values one can assume in the solution of THF the presence of 6AC-(solvent)_1_ complexes and a minor part of the 6AC molecules can exist. In DBE and nitriles the energy of hydrogen-bonding should be high enough to form 6AC-(solvent)_1_ complexes with only a minor part of the 6AC molecules, which are present in the solution.

The above presented predicted values of *E*_HB_ in the S_0_ state for 6AC-(solvent)*_n_*, *n* = 1, 2, complexes, and their changes upon S_0_→S_1_ excitation, together with β Kamlet-Taft’s solvatochromic parameter values, allow us to predict which species can exist in the S_1_ emitting excited state, depending on the solvent. Because of the significant electron density decrease on the hydrogen atoms of the amino group in the excited S_1_ state compared to that in the ground S_0_ state, the energy of C type hydrogen bonds in the S_1_ state must be significantly higher than that in S_0_ state. Therefore, for DMSO and HMPA, almost only 6AC-(solvent)_2_ S_1_-excited complexes have to be present. In the solution of DMF the 6AC-(solvent)_2_ and 6AC-(solvent)_1_ S_1_-excited complexes can coexist, but 6AC-(solvent)_2_ ones dominate. In the case of THF almost all 6AC molecules form 6AC-(solvent)_1_ S_1_-excited complexes. However, only a part of 6AC molecules present in DBE and nitriles solutions can form 6AC-(solvent)_1_ S_1_-excited complexes.

### 2.3. Photophysical Study Results

**Figure 5:**
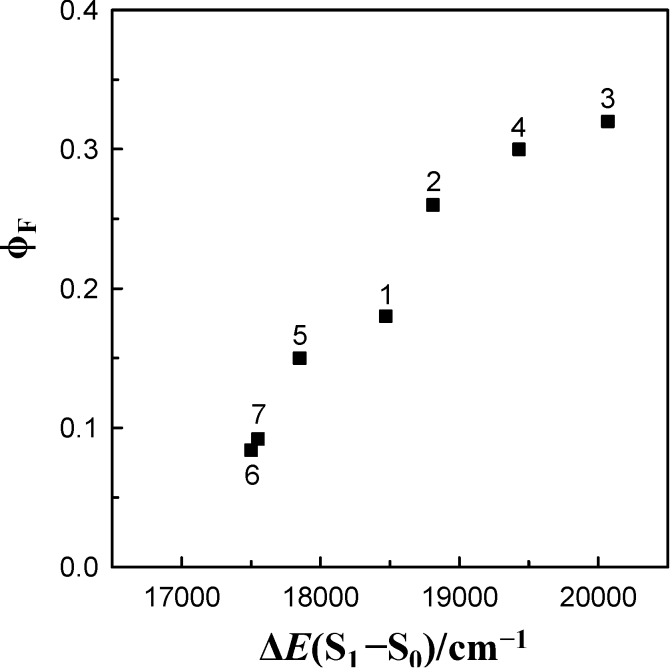
Correlation between the fluorescence quantum yield, Φ_F_, and the ∆*E*(S_1_–S_0_) = 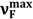 energy gap for 6AC in (1) acrylonitrile, (2) propionitrile, (3) DBE, (4) THF, (5) DMF, (6) DMSO, (7) HMPA.

In order to determine the properties of the species formed by 6AC molecules in their S_1_-excited state in aprotic hydrogen-bond forming solvents, the photophysical measurements were performed (in the same solvents as those used in the spectral studies). Quantum yields of 6AC fluorescence were measured by a relative method using quinine sulphate in 0.05 M H_2_SO_4_ (Φ_F_ = 0.52) [50] as a standard. The Φ_F_ values obtained for 6AC in aprotic hydrogen-bond forming solvents are similar to or lower than those determined for 6AC in 1-chloro-*n*-propane and in other 1-chloro-*n*-alkanes [4], and higher than those obtained for 6AC in protic solvents [5]. The Φ_F_ values increase almost linearly with increasing ∆*E*(S_1_–S_0_) energy gap (see Figure 5), in contrast to C120 [41] and C151 [40]. For these two 7-aminocoumarins, the Φ_F_ values are quite high (~0.5–0.6) in most of the solvents of moderate to high polarities and they do not depend on the solvent. The highest Φ_F_ values for 6AC were obtained in less polar aprotic hydrogen-bond forming solvents (DBE and THF) and the smallest ones in solvents with the highest β Kamlet-Taft solvent parameter.

Fluorescence decay of 6AC in all solvents was usually measured at three wavelengths from the emission spectrum: at the maximum, 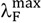, at one wavelength from the short-wavelength side and another one from the long-wavelength side. For 6AC in acrylonitrile, propionitrile, DBE, DMF and DMSO, the fluorescence decays are single-exponential. In THF and HMPA the fluorescence decays of 6AC are found to follow non-single-exponential behaviour, and for them two-exponential function analysis gave reasonably good fits. For 6AC in THF the contribution of the long-time component (τ_1_) component was significantly greater than that of the short-time component (τ_2_). In HMPA two comparable time components (τ_1_,τ_2_) were found, but with significantly different contributions. The values of lifetime components for 6AC in all studied solvents are listed in Table 3. As the fluorescence decays of 6AC in aprotic hydrogen-bond forming solvents are usually single exponential, it is reasonable to assume that the fluorescence lifetimes of the 6AC-(solvent)_1_ and 6AC-(solvent)_2_ S_1_-excited complexes do not differ significantly in a particular solvent. Moreover, they must be similar to the fluorescence lifetime of 6AC in the S_1_-excited state in the same solvent, in analogy to 6AC in protic solvents [5] and to 4-aminophthalimide [1]. Because of the very high energy of hydrogen bonds formed by 6AC molecule with HMPA molecules in the S_0_ state, which are even strengthened due to S_0_→S_1_ excitation, there are practically only 6AC-(HMPA)_2_ S_1_-excited complexes present in HMPA solution in the S_1_-excited state. Therefore, the presence of two lifetime components in fluorescence decay must be associated with two types of emitting 6AC-(HMPA)_2_ complexes having different structures. Notably, these two lifetimes were found at each wavelength from the steady-state emission spectrum range, with the same wavelength independent contributions, which means that their presence in the 6AC decay is not a result of slow solvation.

**Table 3:** Photophysical properties of 6AC in aprotic hydrogen-bond forming solvents.

Solvent	Φ_F_ *^a^*	λ_exc_ (nm)	τ_1_ (ps)	τ_2_ (ps)	*k*_F_ 10^7^ (s^−1^)	*k*_nr_ 10^7^ (s^−1^)
1-Chloro-*n*-propane *^b^*	0.31	367	8200		3.78	8.4
Acrylonitrile	0.18	370	9100		1.98	9.01
Propionitrile *^b^*	0.26	380	12,580		2.07	5.88
DBE	0.32	371	9050		3.53	7.51
THF	0.30	374	13,500 (0.93)	2700 (0.07)	2.22	5.18
DMF	0.15	383	6610		2.27	12.8
DMSO	0.084	387	4800		1.75	19.1
HMPA	0.092	400	6000 (0.80)	4000 (0.20)	1.64	16.2

*k*_F_ = Φ_F_/τ_F_, *k*_nr_ = (1 − Φ_F_)/τ_F_, τ_F_ = τ_1_, for 6AC in HMPA: <τ_F_> = F_1_ τ_1_ + F_2_∙τ_2_ = 5600 ps (F_1_ and F_2_ contributions in parentheses). *^a^* Determined for λ_exc_ corresponding to the maximum of the long-wavelength absorption band with respect to the solution of quinine sulphate in 0.05 mol∙dm^−3^ H_2_SO_4_; *^b^* From reference [4].

As we have shown [4], on the basis of transition dipole moment calculation for 6AC in nitriles (which can form relatively weak hydrogen bonds with the 6AC molecule) the same excited electronic state participates in the absorption and emission processes, in contrast to 6AC in *n*-alkanes and 1-chloro-*n*-alkanes (which interact only non-specifically with the 6AC molecule). Similarly, for 6AC in protic hydrogen-bond forming solvents, the same values of calculated transition dipole moments in absorption and in emission indicate that the same excited electronic state participates in the absorption and emission processes, *i.e.*, S_1_-excited state of the 6AC–(solvent)*_n_*, *n* = 2, 3, complex [5]. Therefore, we have also calculated transition dipole moments for absorption, M_g→e_, and for emission, M_e→g_, for the systems of 6AC in aprotic hydrogen-bond forming solvents, in which the spectral and photophysical studies were performed (see Table 4). For determination of the transition dipole moment modulus squared (CGS), the absorption and emission spectra along with fluorescence decay times and quantum yields were used [51]. Over the range of solvents studied both the absorption transition dipole moments were found not to differ significantly. Moreover, their values are similar in the range of error (±0.2 D) with those of emission transition dipole moments. Since, depending on the solvent, 6AC molecules and/or 6AC–(solvent)*_n_*, *n* = 1, 2, complexes take part in the absorption and emission processes, these three types of species must be characterised by very similar transition dipole moment values.

**Table 4:** 6AC transition dipole moments for absorption (*M*_g→e_) and emission (*M*_e→g_) in aprotic hydrogen-bond forming solvents.

Solvent	*M*_g→e_ (D)	*M*_e→g_ (D)
Propionitrile *^a^*	1.8	2.1
DBE	1.9	2.4
THF	1.9	2.0
DMF	1.9	2.2
DMSO	1.9	1.8
HMPA	1.9	1.8

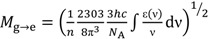
; 
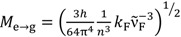
; 
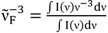
; *k*_F_ = Φ_F_/τ_F_; ε(ν): molar extinction coefficient; *n*: refractive index; *h*: Planck constant; *c*: speed of light; *N*_A_: Avogadro constant; *I*(ν): fluorescence intensity at frequency ν; *^a^* From reference [4].

### 2.4. Deactivation of the Species Formed by 6AC in S_1_-Excited State in Aprotic Hydrogen-Bond Forming Solvents

Analysis of the above presented results of spectral and photophysical studies allows us to propose the deactivation ways of the species present in emitting S_1_-excited state in aprotic hydrogen-bond forming solvents.

Because of the single-exponential decay or a small contribution of τ_2_ component, the radiative, *k*_F_, rate constants were determined using the measured τ_F_ = τ_1_ values. The results are presented in Table 3. The exception was 6AC in HMPA for which <τ_F_> was estimated as defined in the footnote of this Table. Since almost the same influence of solvent polarity and hydrogen-bonding ability as that found for Φ_F_ appears in τ_F_, the *k*_F_ values are not much dependent on the solvent. The only one *k*_F_ value higher, and similar to that for 6AC in 1-chloro-*n*-propane, was obtained for 6AC in DBE, which is characterised by the lowest polarity.

Taking into account a relatively small value of *k*_F_, the nonradiative processes are found to become dominant in S_1_-excited state deactivation of the species present in aprotic hydrogen-bond forming solvents, in contrast to 7-aminocoumarins [40,41]. The nonradiative rate constant values, *k*_nr_, were calculated taking into account the absence of photochemical processes (estimated quantum yield of photochemical decay of 6AC is Φ_PCH_ < 10^−3^ [4]), and they are listed in Table 3. In contrast to *k*_F_, the radiationless deactivation rates are influenced by solvent properties, and *k*_nr_ reaches the highest values for 6AC in the solvents characterised by highest β Kamlet-Taft’s parameter values.

To evaluate the contribution of two possible radiationless processes (internal conversion and intersystem crossing to the triplet state) to photophysical properties of 6AC, we used the results of intersystem crossing quantum yield, Φ_ISC_, measurements for this probe in non-specifically interacting solvents and nitriles [4]. The results presented in [4] clearly show that the Φ_ISC_ value for 6AC in polar nitriles, with which 6AC molecule can form weak hydrogen bonds, is significantly smaller than for 6AC in nonspecifically interacting solvents. The intersystem crossing rate constant value, *k*_ISC_, depends on the solvent and this process becomes less efficient with increasing solvent polarity. Because the polarity of aprotic hydrogen-bond forming solvents used in our study (except for DBE) is only slightly lower than that of acetonitrile, we can assume that the *k*_ISC_ value of 6AC in these solvents is similar to that of 6AC in acetonitrile (*k*_ISC_ = 0.88 × 10^7^ s^−1^) [4]. It has been generally assumed for other aminocoumarins that the triplet formation quantum yield is negligible [40,41,47,52,53]. As seen from Table 3, the *k*_nr_ value determined for 6AC in all aprotic hydrogen-bond forming solvents, is much higher than the assumed value of *k*_ISC_. Therefore the radiationless deactivation rate constant, *k*_nr_, estimated from the equation given in the footnote of Table 3, corresponds essentially to internal conversion, *k*_IC_, thus *k*_nr_ = *k*_IC_, in analogy to 6AC in protic solvents [5]. The internal conversion mechanism involving intermolecular hydrogen-bond complexes was found to be very efficient also e.g., for 2'-aminoacetophenone derivatives [54,55] and fluorenone derivatives [56,57,58,59].

The results shown in Table 1 and Table 3 clearly indicate that *k*_IC_ value depends on the ∆*E*(S_1_–S_0_). There are three possible types of emitting species in aprotic hydrogen-bond forming solvents, *i.e.*, 6AC-(solvent)*_n_*, *n* = 1, 2, S_1_-excited complexes and S_1_-state of 6AC molecule. As previously shown [4], the properties of S_1_-state of 6AC, including τ_F_, depend on the ∆*E*(S_2_(*n*, π*) – S_1_(π, π*)) value, and not on the ∆*E*(S_1_–S_0_) value. In consequence, the ∆*E*(S_1_–S_0_) energy gap law does not describe the non-radiative deactivation rate of S_1_-excited state of 6AC in *n*-alkanes, 1-chloro-*n*-alkanes. Therefore, the clear dependence of Φ_F_~1/τ_F_~*k*_IC_ on the ∆*E*(S_1_–S_0_) suggests that in aprotic hydrogen-bond forming solvents the 6AC-(solvent)*_n_*, *n* = 1, 2, S_1_-excited complexes are the most important species, which are present in the S_1_-excited state and are responsible for the emission process.

**Figure 6:**
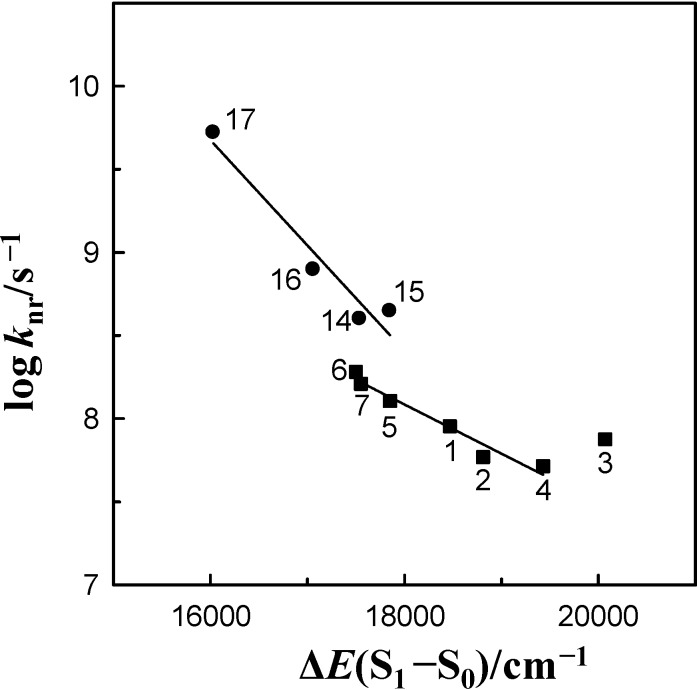
Correlation between the logarithm of the nonradiative deactivation rate constant, log *k*_nr_, and the ∆*E*(S_1_–S_0_) = 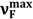 energy gap of 6AC-(solvent)*_n_*, *n* = 1, 2, S_1_-excited complexes in (1) acrylonitrile, (2) propionitrile, (3) DBE, (4) THF, (5) DMF, (6) DMSO, (7) HMPA and of 6AC-(solvent)*_n_*, *n* = 2, 3, S_1_-excited complexes in (14) *^a^* 3,3,4,4,5,5,6,6,6-nonafluorohexanol, (15) *^a^* 1,1,1,3,3,3-hexafluoroisopropanol, (16) *^a^* 2,2,2-trifluoroethanol, (17) *^a^* H_2_O. *^a^* From reference [5].

In Figure 6, the log *k*_nr_ values of 6AC-(solvent)*_n_*, *n* = 1, 2, S_1_-excited complexes in aprotic hydrogen-bond forming solvents and of 6AC-(solvent)*_n_*, *n* = 2, 3, S_1_-excited complexes in protic solvents [5] were plotted against the ∆*E*(S_1_–S_0_) = 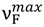 energy gap. This figure demonstrates that the log *k*_nr_ value of these species for 6AC in aprotic hydrogen-bond forming solvents tends to increase linearly (except 6AC in DBE) with decreasing ∆*E*(S_1_–S_0_) and shows that the fast internal conversion is induced by the intermolecular solute-solvent hydrogen-bonding interactions, similarly as in protic solvents [5]. The nonradiative deactivation rate constant to the ground state is generally known to depend exponentially on the energy gap between the excited and ground states [60]. The results of this study, similarly to those in [5], clearly show that the energy gap dependence on radiationless deactivation in an internal conversion process from S_1_-excited state can be observed not only for molecules but also for hydrogen-bonded complexes.

It is easy to notice in Figure 6 that the *k*_nr_ values determined for S_1_-excited complexes in aprotic hydrogen-bond forming solvents are significantly lower (about 2–3 times) than those in protic ones [5], for similar energy gap ∆*E*(S_1_–S_0_) values, *i.e.*, for DMSO, HMPA and 3,3,4,4,5,5,6,6,6-nonafluorohexanol, and for DMF and 1,1,1,3,3,3-hexafluoroisopropanol. Taking into consideration the big energy gap value, it can be assumed that the effective density of the vibrational states in the final electronic state is also independent of the solvent. Therefore, higher *k*_nr_ value in protic solvents must be determined by a higher value of electronic coupling between S_1_ and S_0_ states in the S_1_-excited complexes formed by 6AC with these solvents than with aprotic ones. This follows from the relation: *k*_nr_ = (2π/*h*) *B*^2^ρ*F*, where *B* is the electronic coupling matrix element between the two states, ρ is the effective density of the vibrational states in the final electronic state equiergic with the initially populated state, and *F* is the Frank-Condon factor appropriate energy. For many groups of compounds, a linear correlation between *F* and exp(−∆*E*), where ∆*E* is an energy difference between the lowest vibrational states of the two electronic states has been reported [61,62,63]. To the best of our knowledge this is the first such result for the intermolecular hydrogen-bonded complexes formed by multiatomic molecules of a probe with solvent molecules. Although different linear relationship of *k*_nr_ as a function of ∆*E*(S_1_–S_0_) for 6AC is exhibited between protic and aprotic hydrogen-bond forming solvents (see Figure 6), the slope difference is not very big, as it was affirmed for Coumarin 153 and Coumarin 151 [64]. Similarly to the results obtained for these two coumarins, the slope for 6AC in protic solvents is steeper than that in aprotic ones.

**Figure 7:**
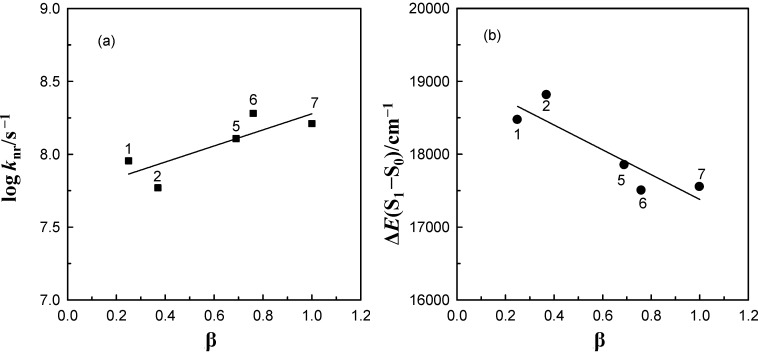
Correlation between the logarithm of the nonradiative deactivation rate constant, log *k*_nr_, (**a**) and the ∆*E*(S_1_–S_0_) = 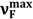 energy gap (**b**) of 6AC-(solvent)*_n_*, *n* = 1, 2, S_1_-excited complexes in acrylonitrile (1), propionitrile (2), DMF, (5), DMSO (6), HMPA (7) and the solvent Kamlet-Taft’s hydrogen-bonding acceptor parameter.

Taking into consideration aprotic hydrogen-bond forming solvents of similar polarity, the log *k*_nr_ values of 6AC-(solvent)*_n_*, *n* = 1, 2, S_1_-excited complexes are correlated well with the Kamlet-Taft’s solvent accepting hydrogen-bond parameter β, supporting the supposition that the degree of hydrogen-bonding of C type between 6AC molecule and solvent molecules directly affects the internal conversion (Figure 7a). Linear relationship between ∆*E*(S_1_–S_0_) and β Kamlet-Taft’s solvent parameter (Figure 7b) clearly show that higher hydrogen bond energy is the cause of energy gap ∆*E*(S_1_–S_0_) decrease.

As indicated above, the results of the study for 6AC in DBE differ significantly from those obtained in other aprotic hydrogen-bond forming solvents. DBE is characterised by a significantly lower polarity. As shown earlier [3,4,5], the spectral and photophysical properties of 6AC molecule are very sensitive to the surrounding environment properties, including polarity and hydrogen-bonding ability of the solvent. As mentioned above, in S_1_-excited state 6AC molecules together with 6AC-(solvent)_1_ complexes are present in the DBE solution. Therefore, the internal conversion of 6AC arises from vibronic interactions between close-lying S_1_(π, π*) and S_2_(*n*, π*) states. Because the polarity of DBE is significantly lower than that of other aprotic hydrogen-bond forming solvents, the triplet state formation from S_1_-excited state of 6AC can be possible, like for 6AC in 1-chloro-*n*-hexadecane [4], whose polarity is comparable to that of DBE.

## 3. Experimental and Computational Methods

6AC was synthesized from hydrochloride (Alfa Aesar) and quantitatively neutralised with an NaOH solution. The compounds: 1-chloropropane, (99%) (Aldrich), 1-chlorobutane (99.5%) anhydrous (Aldrich), 1-chlorohexane (99%) (Aldrich), 1-chlorodecane (98%) (Aldrich), 1-chlorohexadecane (98%) (Fluka), propionitrile (Fluka), acrylonitrile (for organic synthesis) (Synchemica), di-*n*-butyl ether, DBE, (99.3%) (Sigma-Aldrich), tetrahydrofuran, THF, (HPLC) (POCH), *N*,*N*-dimethylformamide, DMF, (spectr.) (Merck), hexamethylphosphoramide, HMPA, (99%) (Aldrich), were additionally dried over molecular sieves (3A, 4A). Anhydrous dimethyl sulphoxide, DMSO, (Aldrich) was used as received.

When necessary, the samples were deoxygenated by bubbling dried oxygen-free helium through them for at least 5 min.

Absorption spectra were measured on a Jasco V-550 spectrometer. Steady-state emission measurements were made on a Jobin Yvon-Spex Fluorolog 3-22 spectrofluorimeter. The emission spectra were corrected for the sensitivity of the detection system. The picosecond laser system and time-correlated single-photon counting (TCSPC) detection systems used to measure fluorescence lifetimes with picosecond precision have been previously described in detail [65,66,67]. Time-per-channel of the setup multi-channel analyser (MCA) was set to 12.2 ps and the fluorescence decays were accumulated in 4000 channels. All measurements were performed at room-temperature.

The energy of the hydrogen bond was calculated as a difference between the sum of the total energies of the isolated 6AC and HMPA molecules and the total energy of the 6AC-HMPA complex. The equilibrium structures of these species were determined using the Moeller-Plesset second-order perturbation method (MP2) with the split-valence basis set 6-31G(d,p). The total energies were then calculated using the MP2 method with the augmented correlation-consistent basis set aug-cc-pVDZ. The counterpoise corrections were not applied.

## 4. Conclusions

It was shown that 6AC can form two hydrogen bonds of donor character between hydrogen atoms of its amino group and aprotic hydrogen-bond forming solvent molecules, which significantly strengthen due to S_0_→S_1_ electronic excitation. These hydrogen bond energy changes between two electronic states were determined experimentally on the basis of spectral absorption and emission measurements.

The results of this study and those presented in [3,4,5,68] make up a complete characterisation of the solvent properties effect on the spectral and photophysical properties of 6AC. They show that the 6AC molecule in the ground S_0_ and electronic excited S_1_ state forms stable complexes with molecules of solvents characterised by hydrogen donor as well as hydrogen acceptor properties. The results of the spectral study of 6AC in solvents of different properties show that the shape of absorption spectra as well as steady-state emission spectra do not differ significantly in 6AC present as molecules (in non-specifically interacting solvents) or as complexes of two different types (in protic and aprotic hydrogen-bond forming solvents). On the other hand, the position of the emission band maxima, 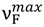, is sensitive to solvent polarity, and similarly as the long wavelength absorption band maxima, 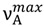, to the hydrogen bonding ability. The photophysical study results clearly show that the intermolecular solute-solvent hydrogen bond formation, irrespective of the hydrogen bond character (donor and acceptor), induces an efficient radiationless deactivation of the S_1_-excited state through internal conversion. As shown for the first time in this study, for hydrogen-bonded complexes there is a linear dependence of the logarithm of the rate constant of nonradiative deactivation in an internal conversion process on the ∆*E*(S_1_–S_0_) energy-gap. Interestingly, the relationship between radiationless deactivation rate constant from S_1_-excited state and the energy gap in aprotic solvents was not much different from that in protic ones [5]. For 6AC in nonpolar aprotic solvents, besides fluorescence, efficient S_1_-ICT→S_0_ internal conversion arises from vibronic interactions between close-lying S_1_-ICT (π, π*) and S_2_ (*n*, π*) states.

The results of our recent theoretical *ab inito* study for 6AC-water complexes [69] are in good agreement with the results of this study and those presented in [3,5] and provide complete results on the solute-solvent hydrogen-bonding effect on the spectral absorption properties of 6AC.

The procedure for independent and accurate determination of excitation or deactivation induced changes in the energy of nonspecific interactions and changes in the energy of particular types of hydrogen bonds proposed by us in [1] has been applied in this paper (as well as in [3,5,70]) for investigation of spectral and photophysical properties of 6AC and the complexes it makes with solvent molecules. The main aim of the study was to establish the effect of solute–solvent hydrogen bonds on the absorption and emission spectral properties and deactivation processes. According to the procedure proposed, a simple analysis of solvatochromic plots provides the information on the origin (type of interaction and change in its energy as a result of excitation or deactivation) of the bathochromic or hypsochromic shifts of bands in the absorption and emission spectra. As the solvatochromic studies are widely used, the procedure for determination of changes in the hydrogen bond energy as a result of excitation or deactivation can be used for any molecules that are able to form different types of hydrogen bonds. Of key importance for successful realisation of the procedure is the proper choice of the solvents for solvatochromic study. We propose the use of 1-chloro-*n*-alkanes as solvents capable of only nonspecific interactions and solvents that are capable of making hydrogen bonds of exclusively acceptor character with the probe, e.g., dimethyl sulphoxide, DMSO, or of exclusively donor character e.g., 1,1,1,3,3,3-hexafluoroisopropanol, HFIP). The advantage of this method is the use of a small number of carefully selected solvents for solvatochromic study.
